# SuccSite: Incorporating Amino Acid Composition and Informative *k*-spaced Amino Acid Pairs to Identify Protein Succinylation Sites

**DOI:** 10.1016/j.gpb.2018.10.010

**Published:** 2020-06-24

**Authors:** Hui-Ju Kao, Van-Nui Nguyen, Kai-Yao Huang, Wen-Chi Chang, Tzong-Yi Lee

**Affiliations:** 1 Department of Computer Science and Engineering, Yuan Ze University, Taoyuan 32003, Taiwan, China; 2 Department of Information Technology, University of Information and Communication Technology, Thai Nguyen 1000, Vietnam; 3 School of Science and Engineering, The Chinese University of Hong Kong, Shenzhen 518172, China; 4 Warshel Institute for Computational Biology, The Chinese University of Hong Kong, Shenzhen 518172, China; 5 Institute of Tropical Plant Sciences, Cheng Kung University, Tainan 701, Taiwan, China

**Keywords:** Protein succinylation, Succinyl group, Substrate specificity, Amino acid composition, *k*-spaced amino acid pair composition

## Abstract

**Protein succinylation** is a biochemical reaction in which a **succinyl group** (-CO-CH2-CH2-CO-) is attached to the lysine residue of a protein molecule. Lysine succinylation plays important regulatory roles in living cells. However, studies in this field are limited by the difficulty in experimentally identifying the substrate site specificity of lysine succinylation. To facilitate this process, several tools have been proposed for the computational identification of succinylated lysine sites. In this study, we developed an approach to investigate the **substrate specificity** of lysine succinylated sites based on **amino acid composition**. Using experimentally verified lysine succinylated sites collected from public resources, the significant differences in position-specific amino acid composition between succinylated and non-succinylated sites were represented using the Two Sample Logo program. These findings enabled the adoption of an effective machine learning method, support vector machine, to train a predictive model with not only the amino acid composition, but also the composition of *k*-spaced amino acid pairs. After the selection of the best model using a ten-fold cross-validation approach, the selected model significantly outperformed existing tools based on an independent dataset manually extracted from published research articles. Finally, the selected model was used to develop a web-based tool, SuccSite, to aid the study of protein succinylation. Two proteins were used as case studies on the website to demonstrate the effective prediction of succinylation sites. We will regularly update SuccSite by integrating more experimental datasets. SuccSite is freely accessible at http://csb.cse.yzu.edu.tw/SuccSite/.

## Introduction

Post-translational modification (PTM) is a chemical form of regulation that occurs after protein translation. This post-translational regulation plays a vital role in a variety of cellular processes including signaling networks, protein degradation, gene transcriptional regulation, protein–protein interaction, and metabolic pathways. The attachment and removal of chemical groups catalyzed by enzymes underlie most PTMs [1]. Protein succinylation is the biochemical reaction in which a succinyl group (-CO-CH2-CH2-CO-) is attached to a lysine residue of a protein molecule. Succinyl coenzyme A (succinyl-CoA) is a cofactor for enzyme-mediated lysine succinylation [2], [3]. Protein lysine succinylation plays important regulatory roles in living cells. For instance, a previous study [2] reported evidence for possible implications of docosahexomic acid (DHA) exposure in the central nervous system. In a related work [4], Kawai et al. demonstrated the ability of DHA to promote succinylation of lysine residues.

Mass spectrometry (MS), a high-throughput biotechnology, is widely utilized to determine a large amount of site-specific succinylated peptides [5], [6]. Due to the labor-intensive experiments of MS-based proteomics in identifying succinylated sites, there is an increasing number of computational tools dedicated to predicting potential succinylated lysine residues for further functional analyses [7], [8], [9], [10]. Succinylation is a site-specific modification that mainly occurs on lysine residues. The process requires substrate specificity—recognition of a succinylated site in accordance with the composition of surrounding residues. Therefore, increasing the precision of succinylation site prediction requires the detailed characterization of substrate specificity.

Table 1 provides a summary of published methods used for the identification of lysine succinylation sites based on protein sequences. For example, a web-based tool developed by Zhao et al. [8] incorporated support vector machine (SVM) with multiple feature-encoding schemes to identify succinylated sites. The iSuc-PseOpt [9] incorporates the random forest algorithm with *k*-nearest neighbors cleaning (KNNC) [11] and the Included Hypothetical Training Samples (IHTS) for the identification of protein succinylation sites. In addition, Xu et al. [7] developed an approach to predict succinylated lysines according to the biochemical property that PTM prefers a specific composition of amino acids around the substrate site. Various features, such as amino acid composition, a flexibility scalar, aromatic content, the net charge, beta entropy, hydrophobic moment, disorder information, and position-specific scoring matrix (PSSM), were also investigated [12]. Despite several approaches and tools for the identification of protein succinylation sites, the number, quality, and performance of datasets were insufficient to meet current demand. Moreover, the recent advancements in high-throughput biotechnologies increased the available data of experimentally verified succinylated sites. Therefore, we were motivated to develop a new approach for identifying protein succinylation sites primarily using the composition of amino acids [13] and the informative *k*-spaced amino acid pairs (KSAAPs) [14]. Additionally, other sequence-based features such as the composition of dipeptides [15] are also taken into account. After the selection of the best model based on the evaluation of cross-validation, the proposed model can provide a better independent testing result than existing online tools. Finally, the proposed model has been adopted to implement a web-based predictor, SuccSite, to accelerate the practical applications for functional proteomics.

**Table 1:** Summary of the training datasets and learning methods of existing tools for the prediction of protein succinylation sites

*Note* : SVM, support vector machine. The method proposed in the current study is highlighted in bold.

## Method

### Data collection and preprocessing

**Figure 1:**
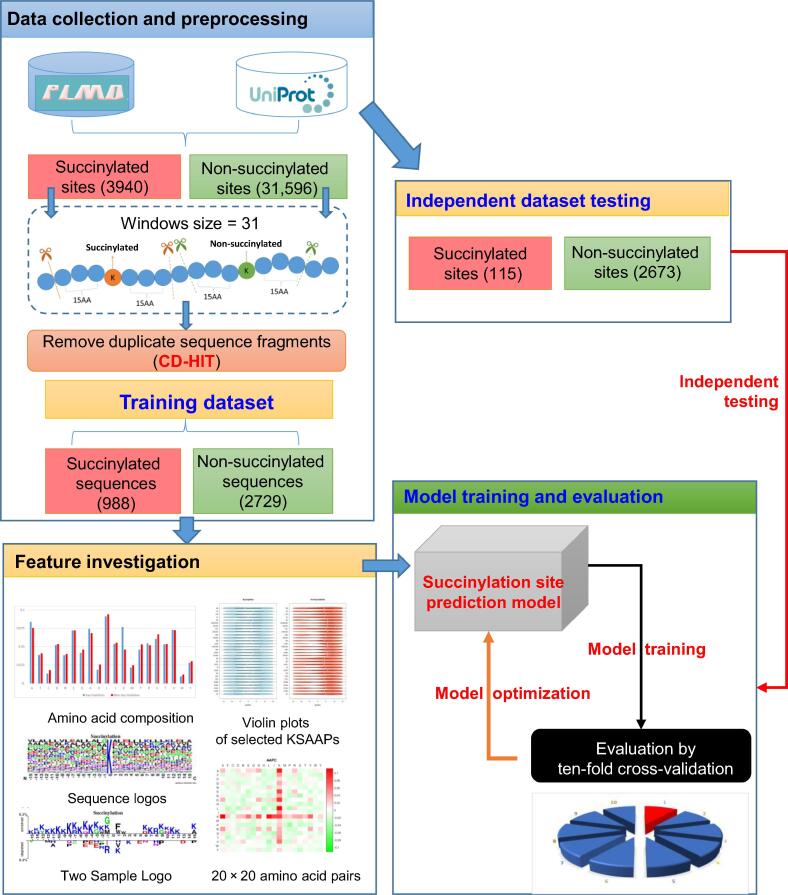
**Flowchart of protein succinylation site prediction in this work**. There are four major steps, including data collection and preprocessing, feature investigation, model training and evaluation, as well as independent dataset testing.

With the advent of high-throughput MS or MS/MS-based proteomics in protein succinylation, numerous resources were developed for compiling experimentally confirmed PTM peptides including lysine succinylated sites based on manual curation of MS/MS-related literature [16], [17], [18]. As presented in Figure 1, the experimentally verified lysine succinylated site dataset was extracted from UniProtKB [19] and CPLM [18]. In UniProtKB, the succinylated proteins are collected and filtered to remove non-experimental entries represented by the Evidence Code Ontology (ECO) codes “0000305”, “0000250”, “0000255”, and “0000256” [20]. This results in 1382 experimentally verified lysine succinylated sites from 419 proteins. The CPLM database 2.0 [18] has 189,919 modified lysines from 45,748 proteins for 12 different types of lysine modifications. With the consideration of only experimentally confirmed lysine succinylated sites, 2558 sites are collected from 897 unique proteins. As a result, 1316 proteins with 3940 experimentally verified lysine succinylated sites are collected from UniProtKB and CPLM. After removing the duplicated and redundant data using the CD-HIT program [21] with a cut-off threshold of 40%, we obtain 1169 unique proteins with 2509 lysine succinylated sites (positive data). In order to prepare the training and independent testing datasets for model training and evaluation, respectively, we randomly select 1000 unique proteins for the training dataset. The remaining 169 unique proteins are the independent testing dataset.

For the identification of lysine succinylation sites, a (*2n + 1*)-mer window size was adopted to extract fragmented sequences centered on modified sites with *n* left-hand and *n* right-hand neighboring amino acids. Given a specific number of succinylated proteins, the negative dataset is generated from non-succinylated sites, which are those fragmented sequences centered on lysine residues without annotation of succinylation. Related works [22], [23], [7], [8], [9], [10] revealed that models trained using a 31-mer window size (*n* = 15) perform best in the prediction of lysine succinylation sites. Owing to the possibility of over-fitting originating from the training dataset, the predictive power of the generated models might be overestimated. Thus, the independent testing dataset, which is blind to the training dataset, is necessary for further evaluation of real cases. In addition, the fragmented sequences may be homologous among datasets used for model training. Therefore, the CD-HIT software is used again to eliminate fragmented sequences with high similarity between the training and testing datasets. As displayed in Table S1, based on a sequence identity threshold of 40%, the final dataset for model training consists of 998 positive and 2729 negative instances; the final dataset for independent testing contained 115 positive and 2673 negative instances. In this work, the positive and negative testing data are further utilized to compare the proposed model with other prediction schemes for predictive performance.

### Amino acid composition

This study focuses on the sequence-based characterization of substrate site specificity for protein succinylation. Amino acid composition (AAC) is a typical attribute used to examine substrate site motifs. AAC determines the probability of amino acids occurring in the flanking region of PTM sites [24]. Given a fragmented sequence *x* with a 31-mer string length, *n_x_*(*k*) is the number of a specific amino acid, *k,* occurring in the fragment, where *k* denotes the 20 amino acids. Consequently, the probability $P_{x}\left(k\right)$ of specific amino acid *k* is [25]

(1) $$P_{x}\left(k\right)=\frac{n_{x}\left(k\right)}{\sum_{k=1}^{20}n_{x}\left(k\right)}\;k=1,2,\cdots ,20$$

Then, the composition of the 20 amino acids can be transformed to a 20-dimensional numeric vector $V_{x}$ for the fragmented sequence *x*:

(2) $$V_{x}=\left[P_{x}\left(1\right),P_{x}\left(2\right),{\cdots ,P}_{x}\left(20\right)\right]$$

In order to observe the position-specific AAC for lysine succinylated sites, WebLogo [26] is utilized to create frequency plots of sequence logos for visualizing the potential amino acid motifs surrounding succinylated sites (at position 0). In addition, a web-based program, Two Sample Logo [27], is adopted to further discover the differences in the position-specific composition of amino acids between succinylated sites (positive data) and non-succinylated sites (negative data).

### Amino acid pair composition

The dipeptides surrounding the succinylated sites are explored by calculating the occurring probability of each amino acid pair (dipeptide) around the substrate sites. The probabilities of 400 dipeptides are compared between succinylated and non-succinylated data to determine the significant dipeptides for model construction. For a fragmented sequence *x*, *f_k_*(*x*) represents the occurrence frequency of a specific amino acid pair. The occurrence frequency of an amino acid pair *pk*(*x*) is defined as follows:

(3) $$pk\left(x\right)=\frac{f_{k}\left(x\right)}{\sum_{i=1}^{400}f_{i}\left(x\right)}\;i,k=1,2,\cdots ,400$$

Then, the composition of the 400 amino acid pairs for a fragmented sequence *x* is

(4) $$P\left(x\right)=\left[p_{1}\left(x\right),p_{2}\left(x\right),{\cdots ,p}_{400}\left(x\right)\right]$$

In order to provide a better observation of amino acid pair composition (AAPC), a 20 × 20 matrix is illustrated with red and green colors to represent over-expression and under-expression of dipeptides, respectively, around succinylated sites. Along with generating sequence logos for lysine succinylated sites and creating the heatmap of AAPC between succinylated and non-succinylated sites, the different value of each amino acid pair as well as its *P* value are determined. All the dipeptides are ranked according to their *P* values, and the dipeptides having a probability difference value >0.02 and a *P* value <0.05 are selected as significant attributes for the classification between positive and negative instances.

### Composition of informative KSAAPs

**Figure 2:**
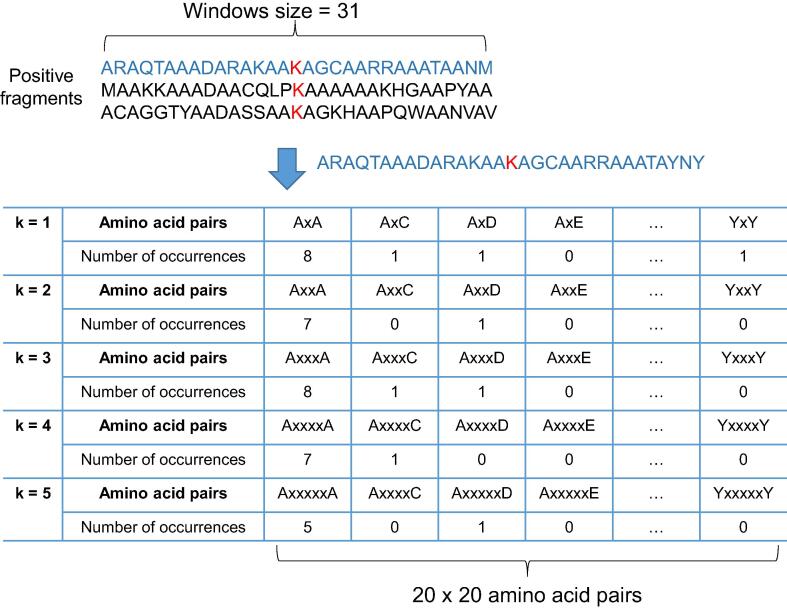
**Composition of k-spaced amino acid pairs**. Given 400 (20 × 20) amino acid pairs and five values for *k* (*k* = 1–5), there are 2000 attributes generated for the CKSAAP feature. The number of occurrences of each k-spaced amino acid pair is determined by sliding through the fragmented sequence one by one. CKSAAP, composition of k-spaced amino acid pair.

In recent years, the composition of k-spaced amino acid pairs (CKSAAPs) [28], [29], [30], represented as a numeric vector in the *n*-dimensional Gaussian space feature, has been widely adopted for the prediction of functional sites on proteins [14], [16], [24], [31], [32], [33], [34], [35], [36]. In this work, we utilize the CKSAAP-based encoding scheme to transform the fragmented sequences into *n*-dimensional numeric vectors. The CKSAAPs are extracted from the flanking amino acid sequences of succinylated sites. As presented in Figure 2, when *k* = 1, $\left[A_{i}xA_{j}\right]$ denotes the pair of amino acids *A_i_* (*i* = 1, …, 20, corresponding to 20 amino acids) and *A_j_* (*j* = 1, …, 20, corresponding to 20 amino acids) that are separated by one amino acid; when *k* = 2, $\left[A_{i}xxA_{j}\right]$ denotes the pair of amino acids *A_i_* and *A_j_* that are separated by two amino acids. Thus, $N(\left[A_{i}xA_{j}\right])$ is the number of occurrences of the one-spaced amino acid pair $\left[A_{i}xA_{j}\right]$ in the training dataset and the conditional probability $P\left[A_{i}xA_{j}\right]$ is

(5) $$P\left[A_{i}xA_{j}\right]=\frac{N(\left[A_{i}xA_{j}\right])}{N(\left[A_{i}xA_{*}\right])},$$

where $N\left(\left[A_{i}xA_{*}\right]\right)=\sum_{j=1,\cdots ,20}N\left(\left[A_{i}xA_{j}\right]\right)$. The strength of the one-spaced amino acid pair [*A_i_xA_j_*] between positive and negative datasets is given by

(6) $$C\left[A_{i}xA_{j}\right]=\log\frac{P^{+}\left[A_{i}xA_{j}\right]}{P^{-}\left[A_{i}xA_{j}\right]},$$

where $P^{+}\left[A_{i}xA_{j}\right]$ and $P^{-}\left[A_{i}xA_{j}\right]$ are the conditional probabilities of the one-spaced amino acid pair $\left[A_{i}xA_{j}\right]$ in positive and negative datasets, respectively. If $C\left[A_{i}xA_{j}\right]>0$, then $\left[A_{i}xA_{j}\right]$ is enriched in the positive dataset; otherwise, the $\left[A_{i}xA_{j}\right]$ is depleted in the positive dataset if $C\left[A_{i}xA_{j}\right]<0$. The high value of $\left|C\left[A_{i}xA_{j}\right]\right|$ indicates that the one-spaced amino acid pair $\left[A_{i}xA_{j}\right]$ is the more significant attribute for classifying between positive and negative datasets. Applying this approach, which is similar to previous work [28], [29], [30], for CKSAAP features, this study has examined the KSAAPs with *k* ranging from one to five. Given 20 × 20 amino acid pairs and five values for *k*, 2000 attributes are used to train the predictive model. However, the higher dimensions of feature vectors could induce a lower efficiency of model learning and evaluation. Therefore, all 2000 features should be tuned to obtain optimal CKSAAPs for providing better predictive performance.

In order to extract informative features prior to the construction of the predictive model, each attribute (*e.g.*, KSAAPs) is evaluated according to the index score calculated by the minimum redundancy–maximum relevance (mRMR) [37] algorithm, which ranks all attributes according to each attribute’s relevance value corresponding to the dataset as well as each attribute’s redundancy index among all 2000 examined KSAAPs. An attribute having maximum relevance and minimum redundancy will contain the best discriminating power between positive and negative instances [38]. The scoring function of mRMR is

(7) $$\mathrm{scor}e_{j}=I\left(f_{j},c\right)-\frac{1}{m}\sum_{i=1}^{m}I\left(f_{i},f_{j}\right)$$

wherein $f_{j}\subset S_{n},\;f_{i}\subset S_{m},\;S_{m}=S-S_{n}$ in which *S_m_*, *S_n_*, and *S* are the attribute sets (*m* and *n* were the attribute sizes). The classification variable *c* stands for two classes corresponding to positive and negative datasets in this work. Additionally, the mutual information $I\left(x,y\right)$ is defined as

(8) $$I\left(x,y\right)=\iint p\left(x,y\right)\log\frac{p(x,y)}{p(x)p(y)}dxdy,$$

where p(x,y), p(x), and p(y) are regarded as the probabilistic density functions between attributes *x* and *y*. Herein, all the KSAPPs were examined by the mRMR criteria. Furthermore, an incremental strategy for extracting useful features, called sequential forward selection (SFS), is applied to conduct a final CIKSAAP with best predictive performance. There are five main steps in this investigation:

1. Choose a classifier (*e.g.*, SVM) and determine an evaluation benchmark (*e.g.*, ten-fold cross-validation).
2. Among all unselected attributes, choose the attribute (KSAAPs) with lowest mRMR index score and combine it into the set of selected attributes.
3. Construct the classifier based on the set of selected attributes.
4. Evaluate the predictive performance of the constructed classifier based on the evaluation benchmark.
5. Repeat steps 2–4 until a sufficient number (default 30) of attributes has been selected, or until predictive performance has been optimized.

### Model construction for succinylation site prediction

This study involves a binary classification between succinylated and non-succinylated sites on lysine residues. The positive and negative datasets are labeled with +1 and −1, respectively, for the two classes. The training dataset is $X=\left\{x^{t},c^{t}\right\}$ where $c^{t}=+1$ if $x^{t}\in$ positive dataset and $c^{t}=-1$ if $x^{t}\in$ negative dataset. Thus, *w* and *w*_0_ were identified such that

$$w^{T}x^{t}+w_{0}\geq +1\;for\;c^{t}=+1\;and\;w^{T}x^{t}+w_{0}\leq -1\;forc^{t}=-1,$$

which can be rewritten as

$$c^{t}\left(w^{T}x^{t}+w_{0}\right)\geq +1$$

This can be used to find an optimal separating hyperplane that can maximize the margin between the two classes [39]. The distance of $x^{t}$ to the discriminating hyperplane is

$$\frac{\left|w^{T}x^{t}+w_{0}\right|}{\left\|w\right\|}$$

and the distance should be higher than a specific value *h*:

$$\frac{c^{t}\left(w^{T}x^{t}+w_{0}\right)}{\left\|w\right\|}\geq h,\;\forall t\;and\;c^{t}\in \left\{+1,-1\right\}$$

The SVM is an advanced algorithm used to identify a hyperplane between two classes with maximum margin based on *n*-dimensional vector space [39] with an attempt to maximize *h*, however, an unlimited number of possible values could be elucidated by tuning *w*. Hence, the h‖w‖ is defined as one to minimize ‖w‖ using the following solution [35]:

$$\min\frac{1}{2}{\left\|w\right\|}^{2}\;subject\;to\;c^{t}\left(w^{T}x^{t}+w_{0}\right)\geq +1,\forall t$$

The SVM can determine a hyperplane for discriminating between succinylated and non-succinylated instances with maximal margin in a vector space containing *n* dimensions (size of attribute set). The sequence-based features are transformed into numeric vectors in an n-dimensional vector space, which are the input values for SVM. A SVM public resource, LIBSVM [40], has been downloaded and installed for iterative training of multiple SVMs in accordance with various feature sets. In the machine learning problem, if the best discriminant is nonlinear, instead of enabling nonlinear modeling, we can map all *n*-dimensional vectors to new vector space with higher dimension *m*, where *m* > *n*, using nonlinear kernel functions. As demonstrated in previous methods [31], [41], [42], [43], [44], the radial basis function (RBF) is typically chosen as the specified kernel function on learning for SVM models. The RBF function is as follows:

$$K\left(x^{t},x\right)=exp\left[-\frac{{\|x^{t}-x\|}^{2}}{2s^{2}}\right]$$

where $x^{t}$ is the center and *s* is the radius, which should be provided by the programmer. When using LIBSVM, cost (*c*) and gamma (*r*) are two supporting parameters used to optimize the radius of kernel function and softness of hyperplane, respectively. To achieve the feasible values of gamma (*r*) and cost (*c*) in model learning, an optimization program, written in Python, was provided by LIBSVM.

### Performance measurement

In this work, the ten-fold cross-validation (10-fold CV) method is performed to measure classifying power of the constructed SVM models. In 10-fold CV, all positive and negative training instances are split into ten subsets with approximately equal data size. After obtaining ten subsets, nine are used as the training dataset, whereas the remaining one subset is used as the test dataset. Each subset, selected from the ten subsets, is regarded as the test dataset until all ten subsets are tested in a 10-fold CV. The performance of the trained models is estimated according to the following metrics:

$$Sensitivity\;\left(Sn\right)=\frac{\mathrm{TP}}{\mathrm{TP}+FN},$$

$$Specificity\;\left(SP\right)=\frac{\mathrm{TN}}{\mathrm{TN}+FP},$$

$$Accuracy\;\left(Acc\right)=\frac{\mathrm{TP}+TN}{\mathrm{TP}+FP+TN+FN},$$

$$Matthews\;correlation\;coefficient\left(MCC\right)=\frac{\left(TP\times TN\right)-\left(FN\times FP\right)}{\sqrt{\left(TP+FN)\times (TN+FP)\times (TP+FP)\times (TN+FN\right)}}$$

in which the predictions of true positives, false negatives, true negatives, and false positives are denoted as TP, FN, TN, and FP, respectively. Accuracy is typically chosen as a benchmark for determining the best predictive model. However, in this investigation, accuracy is not a good benchmark because the size of positive and negative datasets is skewed [36]. Given unbalanced positive and negative datasets in this study, the MCC is used as a reasonable benchmark for taking both prediction rate of TP (sensitivity) and prediction rate of TN (specificity) into account. After 10-fold CV evaluation, the SVM classifier containing the best MCC value is regarded as the best predictor. Finally, a testing dataset, which is independent from the training dataset, is utilized to examine the best model and compare the predicted results with other available online tools.

## Results and discussion

### Composition of amino acids and dipeptides around succinylated sites

**Figure 3:**
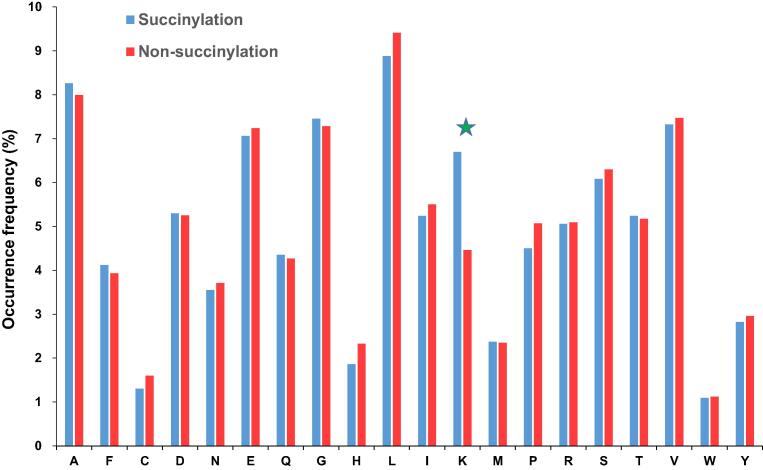
**Comparison of amino acid composition between succinylated sites and non-succinylated sites**. This investigation shows that the positively charged lysine residue (indicated with star) is abundant within the neighborhood of succinylated sites (in blue), compared to non-succinylated sites (in red).

**Figure 4:**
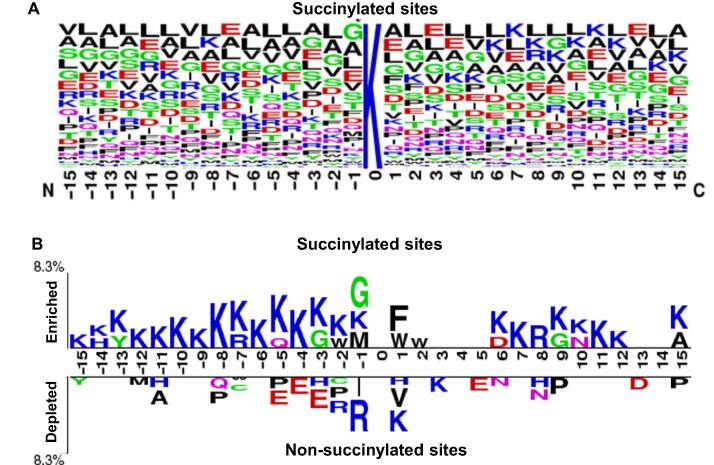
**Position-specific amino acid composition of succinylated sites**. **A.** Frequency plot of substrate sequences. **B.** Two Sample Logo of substrate sequences.

The AAC is a feasible scheme to explore the potential motif of conserved residues around the succinylated sites based on the fragments with 31-mer sequence length. When comparing the AAC between positive and negative datasets, the residues having significant differences are useful attributes for succinylated site prediction. Figure 3 shows that, for succinylated sites, the positively charged lysine residue appears to have the highest frequency around the substrate sites. In addition to AAC, the position-specific AAC neighboring the succinylated sites can be displayed using the frequency plots of WebLogo [26]. As illustrated in Figure 4**A**, no amino acid has significantly high frequency near the succinylated sites, but the slightly prominent amino acid residues include leucine, lysine, alanine, and valine. Without conserved motifs observed in the frequency plot, the Two Sample Logo [27] program was further applied to compare the differences of position-specific AAC between flanking regions of succinylated and non-succinylated sites. As displayed in Figure 4B, the most conserved motifs appear to be associated with charged residues, in particular the positively charged lysine residue at positions −15~−1, +6~+12. In addition, the depletion of negatively charged amino acids, such as glutamic acid, at positions −5, −4, −3, and +5, is predictive. The results reveal that amino acids situated further away in the sequence but closer in the three-dimensional structure to the succinylated sites had notable differences between succinylated sites and non-succinylated sites.

The amino acid pairs surrounding lysine succinylated sites are also explored using the detection of remarkable amino acid pairs with significant differences between positive and negative datasets. In this investigation, a 20 × 20 matrix is adopted to represent the over-expressed and under-expressed amino acid pairs as red and green colors, respectively. As shown in Figure S1, the dipeptides involving a lysine residue in first position, such as KA, KE, KG, and KL, are over-represented around succinylated sites. Interestingly, the dipeptides involving lysine residue in the second position, such as AK, GK, and LK, are also over-represented around succinylated sites. By sorting the amino acid pairs according to their *P* values, the dipeptides with *P* < 0.05 and with a probability difference >2% are extracted and combined into an attribute set with statistical significance.

### Investigation of informative *k*-spaced amino acid pairs

To support the identification of lysine succinylation sites, we have counted and ranked the frequencies of all *k*-spaced amino acid pairs that appeared in the positive and negative training datasets. In this study, top 30 significant KSAAPs, based on sequential forward selection in accordance with their mRMR scores, are selected for the identification of succinylated sites. Figure S2 provides violin plots of selected KSAAPs with their corresponding distributions, ranging from −15 to 15, around succinylated and non-succinylated sites (position 0). This investigation has indicated that most of the selected KSAAPs prefer to locate in the upstream and downstream regions of succinylated sites, whereas in non-succinylated sites these selected KSAAPs have concentrated distribution only in the downstream region. For instance, KA prefers to locate in the upstream and downstream regions of succinylated sites, but for the non-succinylated sites it only prefers to locate in the downstream region. Due to the difference of KSAAPs’ distributions between succinylated and non-succinylated sequences, these top 30 KSAPPs are then incorporated into the construction of SVM models.

### Cross-validation evaluation of characteristics flanking succinylated sites

To obtain the optimal window lengths that generate the best performance, we have investigated and assessed various window lengths using 10-fold CV. In accordance with the difference of position-specific AACs between succinylated and non-succinylated sites as well as our preliminary evaluation, the window size of 31 (−15 ~ +15; with the centered residue at lysine) provides the best performance in the prediction. Based on the investigated features, the corresponding SVM models are built to determine the effectiveness of those features in the identification of succinylated sites. As displayed in Table S2, the AAC-based SVM model has 64.6% accuracy and an MCC value of 0.27. The AAPC-based SVM model yields 63.2% accuracy and an MCC value of 0.24. In addition, the CKSAAP-based SVM model (*K* = 5) obtains 61.9% accuracy and an MCC value of 0.22.

In a binary classification between succinylated and non-succinylated sites, it is feasible to incorporate two or more different attribute sets in modeling. Therefore, in addition to the single attribute set, a hybrid combination of different attribute sets are also considered in this study. Based on the three attribute sets that were investigated (AAC, AAPC, and CKSAAP), four combinations (“AAC + AAPC”, “AAC + CIKSAAP”, “AAPC + CIKSAAP”, and “AAC + AAPC + CIKSAAP”) are analyzed for the identification of succinylated sites. Table S2 presents the performance of the hybrid features-based models when evaluated using 10-fold CV. The results reveal that most hybrid features-based models can obtain better performance, wherein the “AAC + CIKSAAP”-based model performs the best, with 71.4% accuracy and an MCC value of 0.40. Thus, the hybrid features of AAC and CIKSAAP yield the most promising predictions. In addition, the ROC curve is generated to compare different predictive models. As displayed in Figure S3, the results show the SVM model trained from the combination of AAC and CKSAAP attribute sets gave the best predictive power.

### Evaluation of the selected models using independent test set

**Figure 5:**
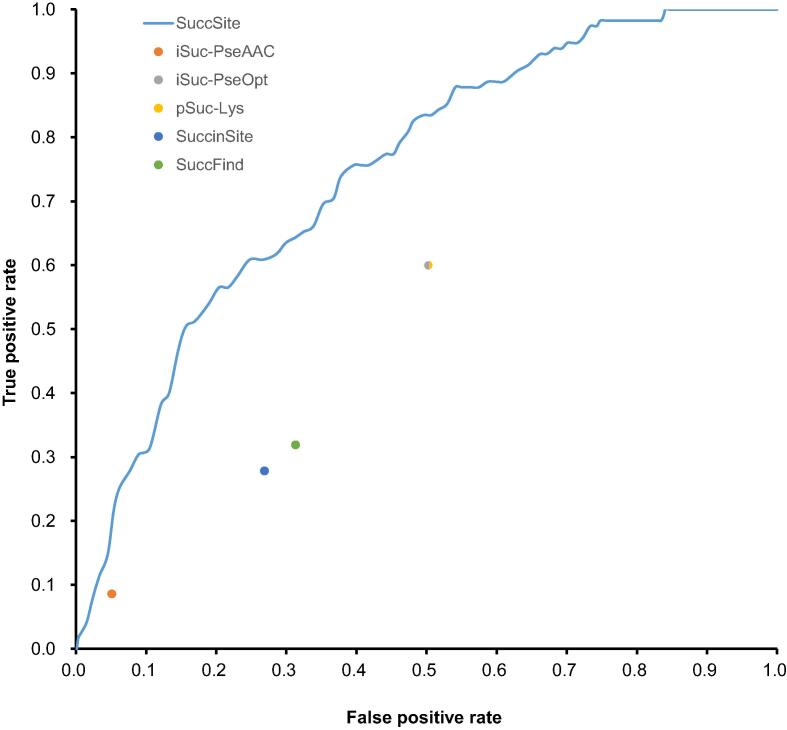
**Comparison of ROC curves between SuccSite and other succinylation site prediction tools**.

In the classification between positive and negative instances, there is a potential risk to over-estimate the predictive performance because of over-fitting in model training. Therefore, an independent testing dataset was constructed to assess the model’s ability and stability in practice. As mentioned previously, the independent testing dataset comprised 115 positive and 2673 negative sites. To assess the practical ability of our proposed model, the comparison between our model and several existing prediction tools is performed using the testing dataset. As displayed in Table 2 and Figure S4, our proposed model achieves higher values on both accuracy and MCC value, reaching 82.9% accuracy and a MCC value of 0.18. In addition, to provide an overview of the models’ predictive ability, ROC curves are used to compare our proposed model with existing succinylation sites prediction tools in independent testing. As displayed in Figure 5**,** our proposed model outperforms other available prediction tools.

**Table 2:** Performance comparison of SuccSite and other existing tools using an independent testing dataset

### A web-based predictor of the proposed method

**Figure 6:**
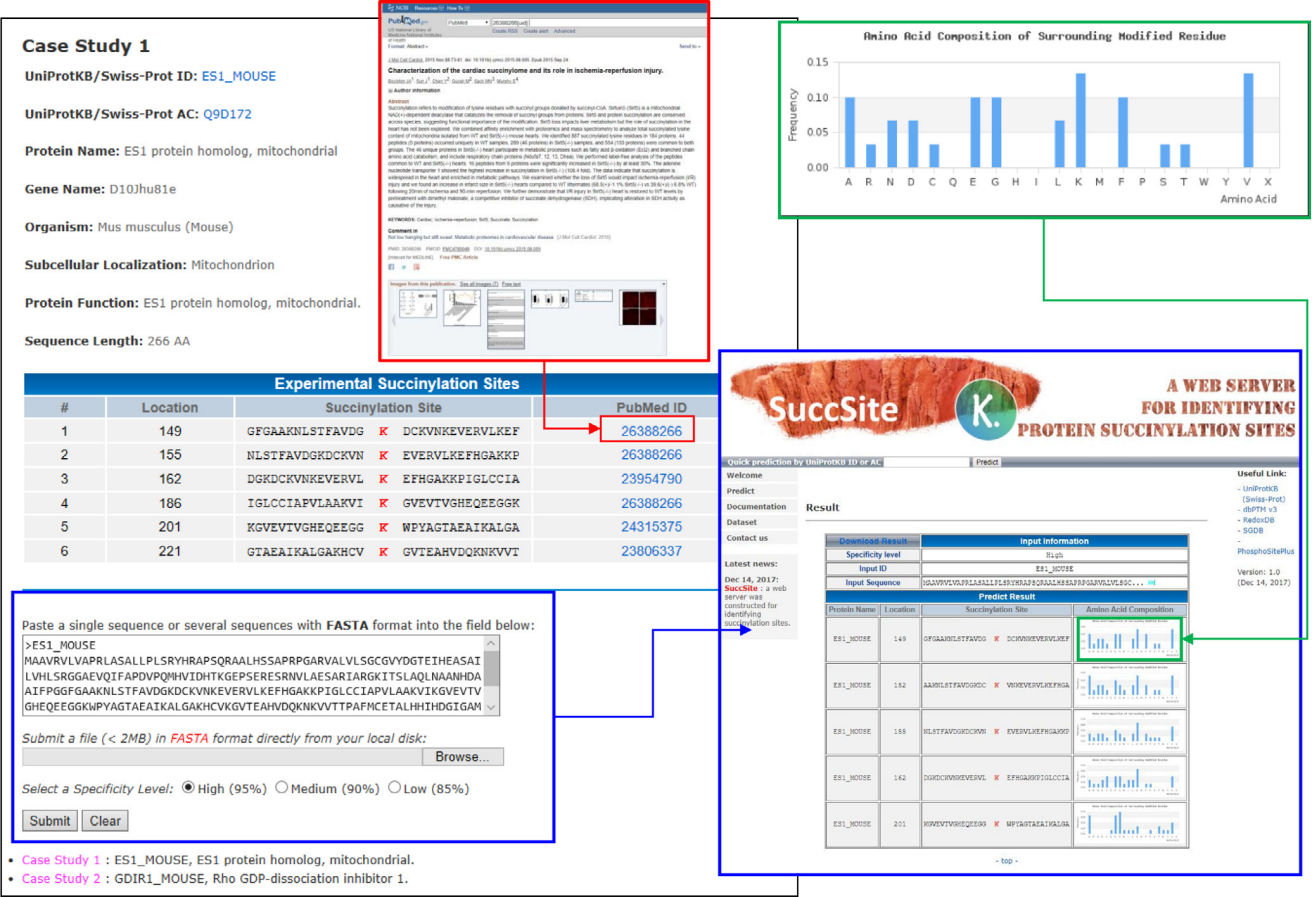
**A case study of succinylation site prediction on ES1 protein homolog, mitochondrial**. The prediction result includes the predicted positions of succinylation sites, flanking amino acids (from −15 to 15), and amino acid composition.

An effective prediction tool can help biologists save time and accelerate the functional study of protein succinylation sites. In this work, a web-based predictor (called SuccSite) is designed for users to analyze protein succinylation sites efficiently. Figure S5 shows the main functions such as predict, documentation, and dataset of SuccSite. Figure 6 shows the prediction information (the prediction results with a bar-chart of AAC for each fragmented sequence having succinylated sites). To demonstrate the effectiveness of the SuccSite predictor, two case studies are provided on the website. The first case study predicts succinylation sites for the ES1 protein homolog, mitochondrial (UniProtID: ES1_MOUSE). The ES1 consists of 266 AA residues, including 19 lysine residues. Six lysine residues are experimentally verified as succinylated substrate sites at positions 149, 155, 162, 186, 201, and 221. As displayed in Figure 6, the SuccSite can predict five succinylation sites at positions 149, 152, 155, 162, and 201. However, position 152 has not yet been experimentally confirmed as a succinylated site. Hence, the estimating TP, FN, FP, and TN of the SuccSite are 4, 2, 1, and 12, respectively. The SuccSite can achieve 84% accuracy, 67% sensitivity, and 92% specificity for this case study. The second case study is the prediction of Rho GDP-dissociation inhibitor 1 (UniProt ID: GDIR1_MOUSE), which consists of 204 AA residues, including 19 lysine residues. Two lysine residues are experimentally verified as succinylated substrate sites at positions 52 and 141. The SuccSite can predict a succinylation site at 141. Hence, the estimating TP, FN, FP, and TN were 1, 1, 0, and 17, respectively. The SuccSite yields 95% accuracy and 100% specificity for this case study.

With reference to the case study of Wang et al. [45], the computational identification of the top 10 potential succinylation sites has been conducted to determine novel succinylated lysines for biochemical communities. This investigation reveals that these potential succinylation sites occur in different proteins. As displayed in Table S3, the potential substrate site having the highest score (0.720) is at lysine 167 of histone H1 protein (UniProt ID: I7HFT9_MOUSE). Interestingly, this site is succinylated, as reported in a previous study [46]. The literature evidence indicates the reliability of the proposed method, SuccSite.

## Conclusion

This work develops a new predictor, SuccSite, to investigate and identify lysine succinylation sites based on AAC and CIKSAAPs. Pipelined analyses of various attributes in the neighborhood of succinylated sites are performed on the large-scale succinyl-proteome data. The Two Sample Logo investigation has revealed that the most remarkable finding is the enrichment of lysine residues within the flanking regions of succinylated sites. According to the 10-fold CV evaluation, the proposed method could yield a promising performance. The independent testing performed demonstrates that the selected SVM model (AAC + CIKSAAP) is comparable to other existing prediction tools. We believe that our proposed approach will help facilitate the determination of succinylated targets on lysine residues of proteins. In addition, to support research involved in the characterization of lysine succinylated sites, a web-based tool named SuccSite has been designed and implemented. The SuccSite is free for use and will be updated regularly.

## Availability

SuccSite is available at http://csb.cse.yzu.edu.tw/SuccSite/.

## Authors’ contributions

HJK and VNN carried out the data collection and curation, participated in the bioinformatics analyses, and drafted the manuscript. HJK and KYH carried out the web tool implementation. WCC participated in the design of the study and performed the draft revision. TYL conceived of the study, and participated in its design and coordination and helped to revise the manuscript. All authors read and approved the final manuscript.

## Competing interests

The authors have declared no competing interests.

## Supplementary material

Supplementary Figure S1 Difference in frequencies among the 20 × 20 amino acid pairs between succinylated sites and non-succinylated sites. Dipeptides in red represent a high abundance in the positive dataset, while those in green represent a high depletion in the positive dataset.

Supplementary Figure S2 Violin plots of selected k-spaced amino acid pairs. Violin plots provide an overall observation of the distribution of a k-spaced amino acid pairs in the flanking region, ranging from -15 to 15, of succinylated and non-succinylated sites (position 0).

Supplementary Figure S3 Comparison of ROC curves among the models trained with various attribute sets.

Supplementary Figure S4 Performance comparison between SuccSite and other tools using independent testing dataset.

Supplementary Figure S5 Front page of SuccSite for predicting lysine succinylation sites on proteins.
