# Supplementary material for: SuccSite: Incorporating Amino Acid Composition and Informative k-spaced Amino Acid Pairs to Identify Protein Succinylation Sites
Source: Genomics Proteomics Bioinformatics. 2020 Jun 24;18(2):208–19. doi: 10.1016/j.gpb.2018.10.010 (PMC7647693; doi:10.1016/j.gpb.2018.10.010)
Supplement: Supplementary Figure S1 — Difference in frequencies among the 20 × 20 amino acid pairs between succinylated sites and non-succinylated sites. Dipeptides in red represent a high abundance in the positive dataset, while those in green represent a high depletion in the positive dataset. [file mmc1.pptx]

## Slide 1
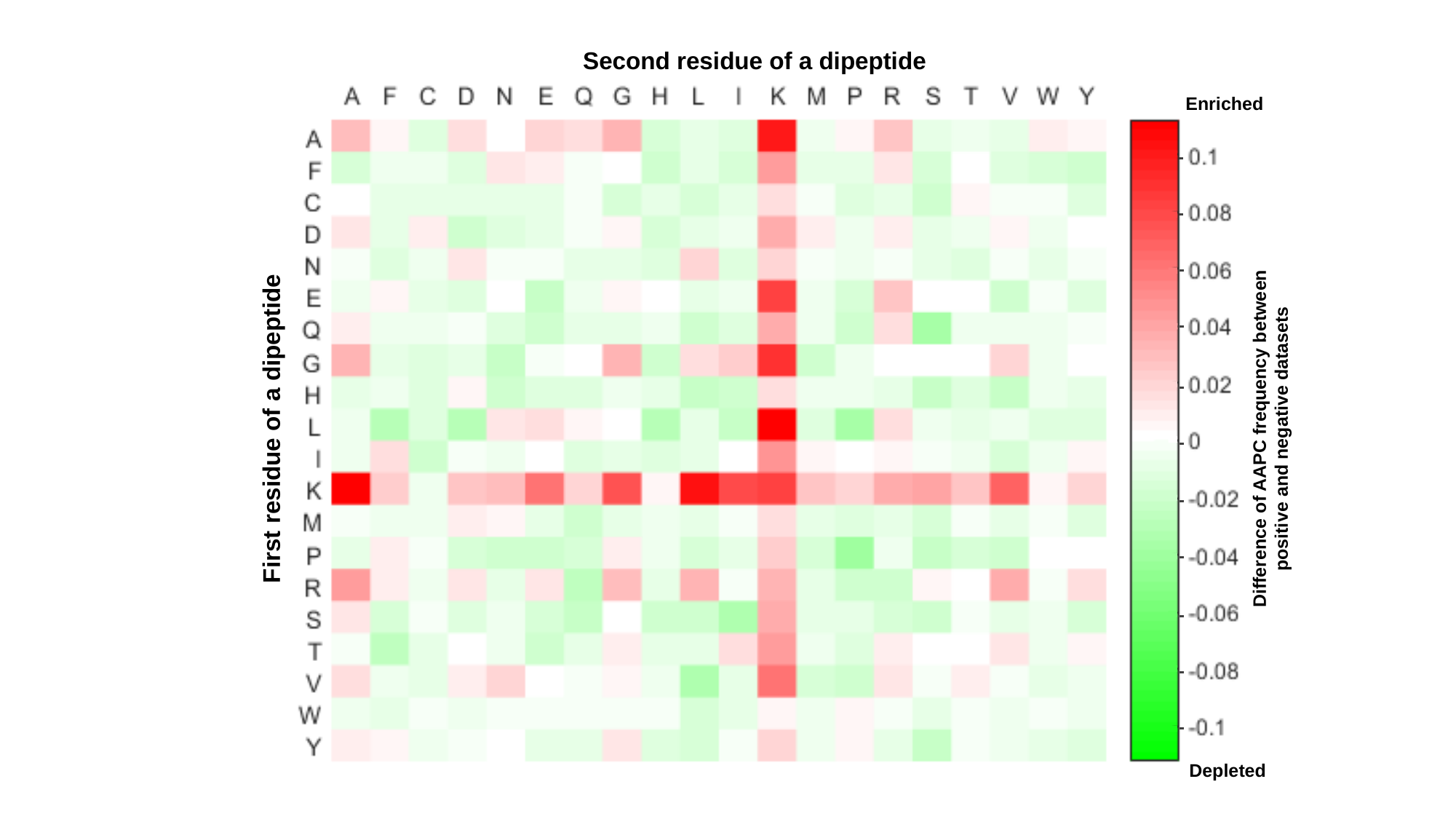

Second residue of a dipeptide
Enriched
First residue of a dipeptide
Difference of AAPC frequency between positive and negative datasets
Depleted
