# Supplementary figures and images for: SuccSite: Incorporating Amino Acid Composition and Informative k-spaced Amino Acid Pairs to Identify Protein Succinylation Sites
Source: Genomics Proteomics Bioinformatics. 2020 Jun 24;18(2):208–19. doi: 10.1016/j.gpb.2018.10.010 (PMC7647693; doi:10.1016/j.gpb.2018.10.010)

## Slide 1
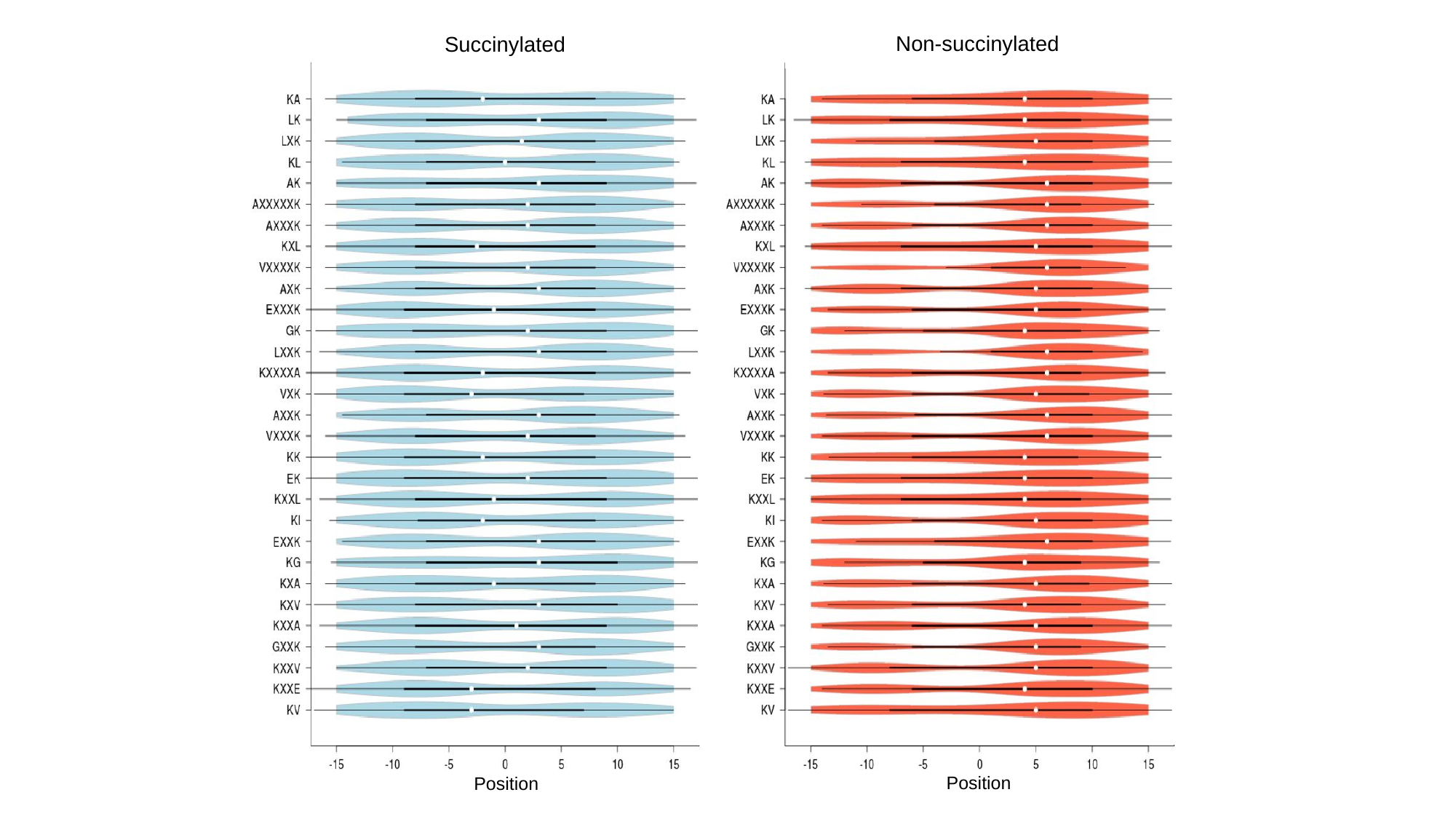

Non-succinylated
Succinylated
Position
Position

Supplement: Supplementary Figure S2 — Violin plots of selected k-spaced amino acid pairs. Violin plots provide an overall observation of the distribution of a k-spaced amino acid pairs in the flanking region, ranging from -15 to 15, of succinylated and non-succinylated sites (position 0). [file mmc2.pptx]

## Slide 1
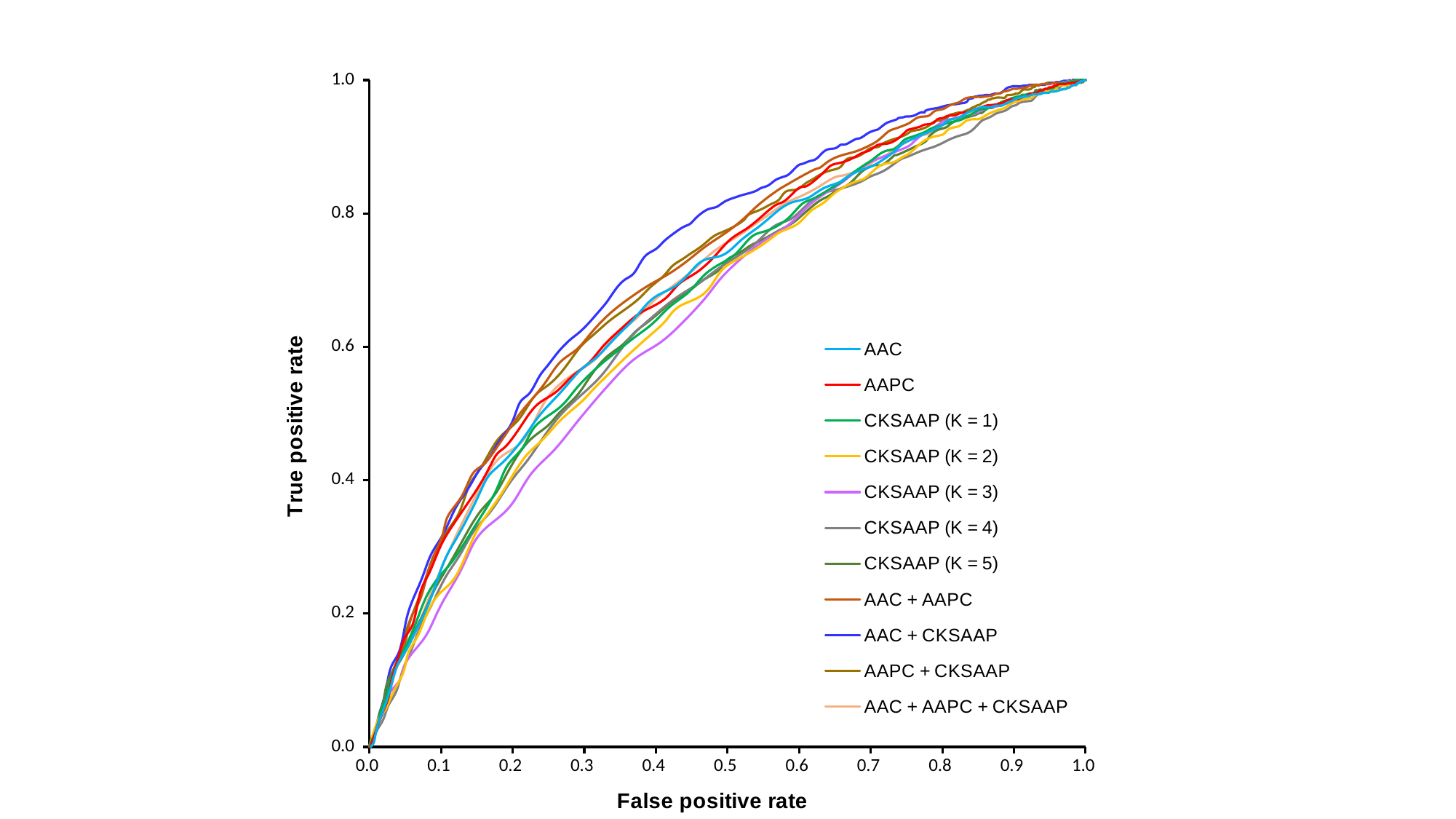

### Chart
| Category | | | | | | | | | | | |
|---|---|---|---|---|---|---|---|---|---|---|---|

Supplement: Supplementary Figure S3 — Comparison of ROC curves among the models trained with various attribute sets. [file mmc3.pptx]

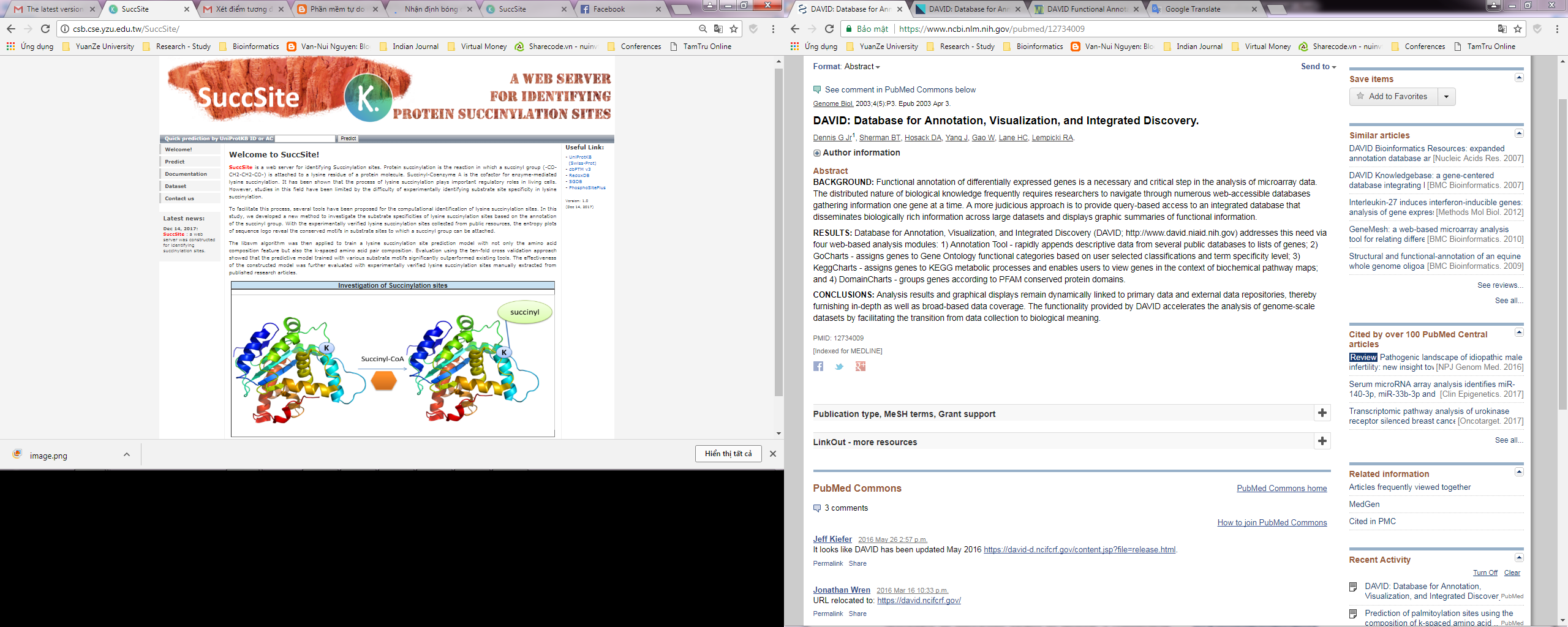

Supplement: Supplementary Figure S5 — Front page of SuccSite for predicting lysine succinylation sites on proteins. [file mmc5.docx]
