# Supplementary material for: SuccSite: Incorporating Amino Acid Composition and Informative k-spaced Amino Acid Pairs to Identify Protein Succinylation Sites
Source: Genomics Proteomics Bioinformatics. 2020 Jun 24;18(2):208–19. doi: 10.1016/j.gpb.2018.10.010 (PMC7647693; doi:10.1016/j.gpb.2018.10.010)
Supplement: Supplementary Figure S4 — Performance comparison between SuccSite and other tools using independent testing dataset. [file mmc4.pptx]

## Slide 1
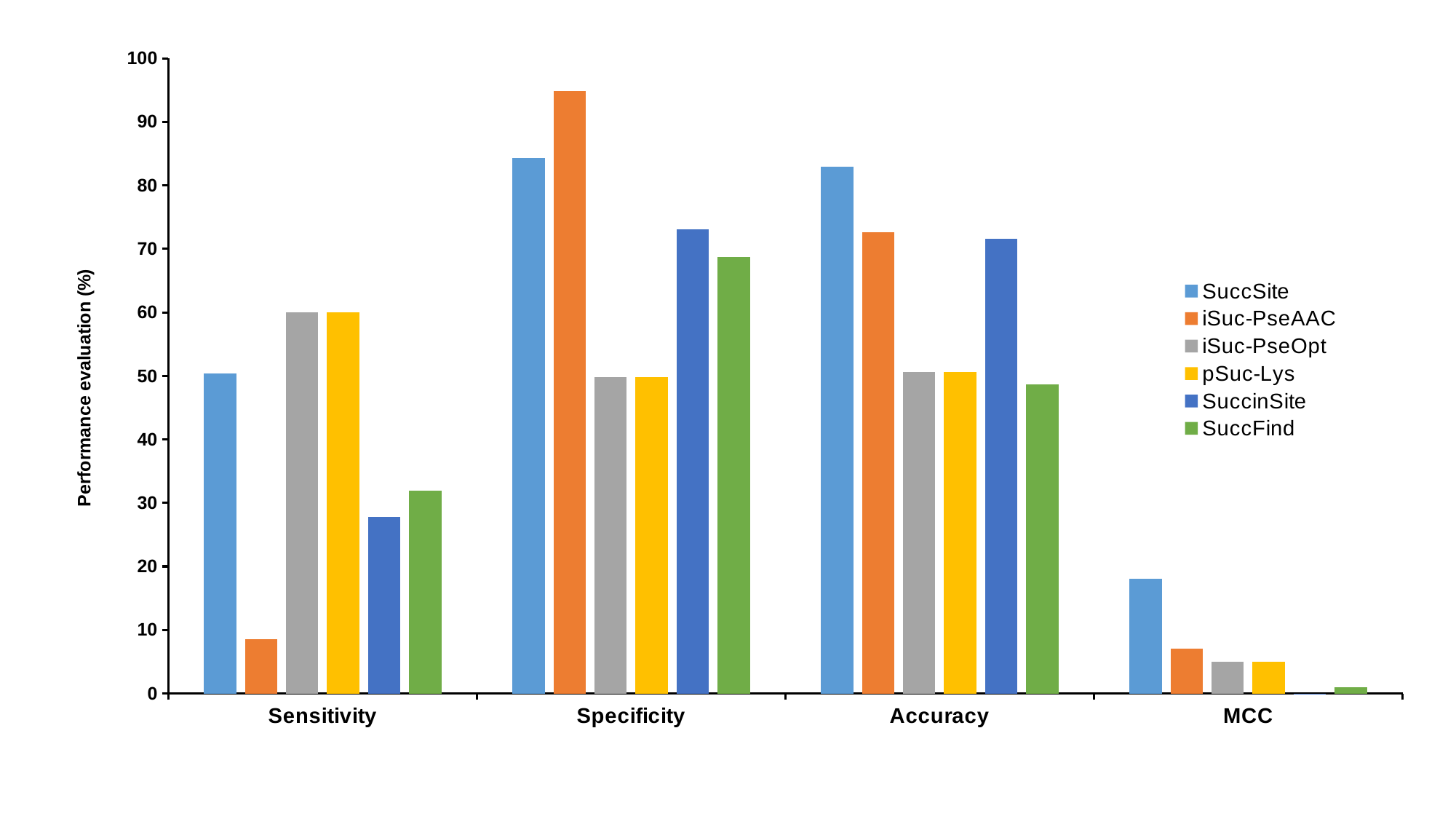

### Chart
| Category | SuccSite | iSuc-PseAAC | iSuc-PseOpt | pSuc-Lys | SuccinSite | SuccFind |
|---|---|---|---|---|---|---|
| Sensitivity | 50.43 | 8.6 | 60.0 | 60.0 | 27.826086956521692 | 31.9 |
| Specificity | 84.32 | 94.9 | 49.77941176470591 | 49.77941176470591 | 73.1324544883867 | 68.7 |
| Accuracy | 82.93 | 72.6 | 50.5762711864407 | 50.5762711864407 | 71.55407452287187 | 48.7 |
| MCC | 18.0 | 7.0 | 5.0 | 5.0 | 0.003963670619334504 | 1.0 |
