# Supplementary material for: SuccSite: Incorporating Amino Acid Composition and Informative k-spaced Amino Acid Pairs to Identify Protein Succinylation Sites
Source: Genomics Proteomics Bioinformatics. 2020 Jun 24;18(2):208–19. doi: 10.1016/j.gpb.2018.10.010 (PMC7647693; doi:10.1016/j.gpb.2018.10.010)
Supplement: Supplementary Table 1 [file mmc6.docx]

**Table S1 Data statistics of removing homologous sequences by using CD-HIT with various identity thresholds**

| **Threshold** | **No. of succinylated proteins** | **No. of succinylated lysine sites** | **No. of non-succinylated lysine sites** |
| --- | --- | --- | --- |
| Raw data | 1316 | 3940 | 31,596 |
| 100% | 1229 | 3591 | 27,919 |
| 90% | 1224 | 3453 | 22,620 |
| 80% | 1222 | 3233 | 19,222 |
| 70% | 1215 | 2996 | 16,565 |
| 60% | 1205 | 2855 | 14,567 |
| 50% | 1197 | 2701 | 13,047 |
| 40% | 1169 | 2509 | 11,078 |
| 30% (final dataset) | 758 | 1103 | 5402 |
| Training dataset | 643 | 988 | 2729 |
| Independent dataset | 115 | 115 | 2673 |
