# Supplementary material for: SuccSite: Incorporating Amino Acid Composition and Informative k-spaced Amino Acid Pairs to Identify Protein Succinylation Sites
Source: Genomics Proteomics Bioinformatics. 2020 Jun 24;18(2):208–19. doi: 10.1016/j.gpb.2018.10.010 (PMC7647693; doi:10.1016/j.gpb.2018.10.010)
Supplement: Supplementary Table S2 [file mmc7.docx]

**Table S2 Performance evaluation using ten-fold cross-validation on various attribute sets**

| **Feature** | **Sensitivity** | **Specificity** | **Accuracy** | **MCC** |
| --- | --- | --- | --- | --- |
| AAC | 67.4% | 63.5% | 64.6% | 0.27 |
| AAPC | 64.7% | 62.7% | 63.2% | 0.24 |
| CKSAAP (K = 1) | 63.6% | 60.4% | 61.2% | 0.21 |
| CKSAAP (K = 2) | 61.7% | 60.8% | 61.0% | 0.20 |
| CKSAAP (K = 3) | 60.5% | 59.5% | 59.8% | 0.18 |
| CKSAAP (K = 4) | 64.4% | 60.6% | 61.6% | 0.22 |
| CKSAAP (K = 5) | 63.7% | 61.2% | 61.9% | 0.22 |
| AAC + AAPC | 69.2% | 68.1% | 68.4% | 0.34 |
| **AAC + CKSAAP** | **73.7%** | **70.6%** | **71.4%** | **0.40** |
| AAPC + CKSAAP | 70.7% | 69.2% | 69.6% | 0.37 |
| AAC + AAPC + CKSAAP | 65.1% | 68.0% | 68.2% | 0.33 |

*Note*: AAC, amino acid composition; CKSAAP, composition of k-spaced amino acid pair; AAPC, amino acid pair composition; MCC, Matthews ccorrelation ccoefficient. The feature with the best performance is highlighted in bold.
