# Supplementary material for: SuccSite: Incorporating Amino Acid Composition and Informative k-spaced Amino Acid Pairs to Identify Protein Succinylation Sites
Source: Genomics Proteomics Bioinformatics. 2020 Jun 24;18(2):208–19. doi: 10.1016/j.gpb.2018.10.010 (PMC7647693; doi:10.1016/j.gpb.2018.10.010)
Supplement: Supplementary Table S3 [file mmc8.docx]

**Table S3 List of top 10 potential succinylation sites identified by SuccSite**

| **Rank** | **UniProt ID** | **Position** | **Flanking sequences of succinylation site** | **Score** |
| --- | --- | --- | --- | --- |
| 1 | I7HFT9_MOUSE | 167 | KKPKATPTKASGSGR **K** TKGAKGVQQRKSPAK | 0.720 |
| 2 | I7HFT9_MOUSE | 144 | AKAKKMGLPRASRSP **K** SSKTKAVKKPKATPT | 0.699 |
| 3 | IQGA2_MOUSE | 1524 | DIIATEDMGIFDVRS **K** FLGVEMEKVQLNIQD | 0.669 |
| 4 | TLN1_MOUSE | 1062 | PLEMDSALSVVQNLE **K** DLQEIKAAARDGKLK | 0.641 |
| 5 | UTP11_MOUSE | 69 | LRKKALEKNPDEFYY **K** MTRAKLQDGVHIFKE | 0.636 |
| 6 | SYNC_MOUSE | 255 | QLNNRHMMIRGENMS **K** ILKARSMITRCFRDH | 0.618 |
| 7 | A2A654_MOUSE | 1327 | SVESDLDARISEPAG **K** GLELSQTKTEVTDSS | 0.611 |
| 8 | UGGG1_MOUSE | 1201 | SPPDANDVVVILNNF **K** SKIIKVKVQKKADMA | 0.604 |
| 9 | E9Q616_MOUSE | 5498 | SKIKMPKHIFSKSKG **K** GGVTGSPEASISGSK | 0.603 |
| 10 | H1BP3_MOUSE | 53 | HVEYQILVVTRLAVF **K** SAKHRPEDVVQFLVS | 0.598 |
